# Hydroclimatic changes and drivers in the Sava River Catchment and comparison with Swedish catchments

**DOI:** 10.1007/s13280-015-0641-0

**Published:** 2015-03-10

**Authors:** Lea Levi, Fernando Jaramillo, Roko Andričević, Georgia Destouni

**Affiliations:** 1 Department of Sustainable Development, Environmental Science and Engineering (SEED), Royal Institute of Technology (KTH), Teknikringen 76, 100 44 Stockholm, Sweden; 2 Department of Physical Geography, Stockholm University, 106 91 Stockholm, Sweden; 3 Bolin Centre for Climate Research, Stockholm University, 106 91 Stockholm, Sweden; 4 Department of Applied Hydraulics, Faculty of Civil Engineering, Architecture and Geodesy, University of Split, 21 000 Split, Croatia

**Keywords:** Hydroclimatic change, Evapotranspiration, Runoff variability, Land-use, Hydropower, Sava River

## Abstract

In this study, we investigate long-term hydroclimatic changes and their possible relation to regional changes in climate, land-use and water-use over the twentieth century in the transboundary Sava River Catchment (SRC) in South Eastern Europe. In a hydropower dominated part of the SRC, unlike in an unregulated part, we find increase in average annual evapotranspiration and decrease in temporal runoff variability, which are not readily explainable by observed concurrent climate change in temperature and precipitation and may be more related to landscape-internal change drivers. Among the latter investigated here, results indicate hydropower developments as most closely related to the found hydroclimatic shifts, consistent with previous such indications in studies of Swedish hydropower catchments. Overall, the present results have quantitatively framed the recent history and present state of hydroclimate in the SRC, of relevance for water resources in several countries and for a majority of their populations. This provides a useful basis for further assessment of possible future hydroclimatic changes, under different scenarios of climate change and land/water-use developments in the region.

**Electronic supplementary material:**

The online version of this article (doi:10.1007/s13280-015-0641-0) contains supplementary material, which is available to authorized users.

## Introduction

Growing concerns about and needs to plan for availability, quality, and sustainable use of freshwater require good understanding of past and present conditions, detection of changes and identification of the causes and possible long-term consequences of changing water resources. Water conditions in the world’s land areas interact constantly with natural and anthropogenic climate change (Hamlet and Lettenmaier 1999; Christensen et al. 2004; Nilsson et al. 2005; Seneviratne et al. 2006; Poff et al. 2007; Dyurgerov et al. 2010, Botter et al. 2013). In addition, the conditions of water on land are also affected by direct anthropogenic changes in land-use (e.g., expansion and/or intensification of agriculture, irrigation expansion, deforestation) and water-use (e.g., water diversions for irrigation, decrease in surface water area due to water diversions, water system modifications related to hydropower expansion) in the landscape itself (Gordon et al. 2005; Botter et al. 2010; Naik and Jay 2011; Destouni et al. 2013; Montanari et al. 2013; Jaramillo and Destouni 2014).

Impacts of climate change on water resources and their management have been recognized and studied for some time (Milly et al. 2005; Darracq et al. 2005; Groves et al. 2008; Kundzewicz et al. 2008; Wisser et al. 2010; Jarsjö et al. 2012). Studies of the water resource effects of changes in human land/water-use have shown that these may be as large as the climate-driven effects, for instance, on evapotranspiration and runoff fluxes (Shibuo et al. 2007; Cuo et al. 2009; Asokan et al. 2010; Kvalevåg et al. 2010; Niyogi et al. 2010; Jarsjö et al. 2012; Sorooshian et al. 2012; Destouni et al. 2013). For example, major water diversions for new irrigation schemes in the Aral Sea drainage basin have increased water losses to the atmosphere by increasing evapotranspiration from irrigated agricultural areas, thereby decreasing runoff to the Aral Sea and causing its dramatic shrinkage from the mid twentieth century until present time (Shibuo et al. 2007; Destouni et al. 2010). These historic land- and water-use changes will also continue to condition future freshwater responses to forthcoming climate change in the region (Jarsjö et al. 2012). Furthermore, Degu et al. (2011) have shown that large dams in various parts of the United States affect available potential energy, surface evaporation, and specific humidity in the atmosphere over distances of up to 100 km from the water reservoirs. Several other recent studies have also reported direct impacts of such anthropogenic changes in land-use and water-use on regional climate (Lobell et al. 2009; Asokan et al. 2010, Destouni et al. 2010; Hossain 2010; Lee et al. 2011; Degu et al. 2011; Asokan and Destouni 2014). In order to improve our predictive capability for future hydroclimatic changes, there is a need to increase understanding of water changes and their main drivers by further studies of such change manifestations under climatic and land/water-use conditions prevailing in different world regions.

**Fig. 1:**
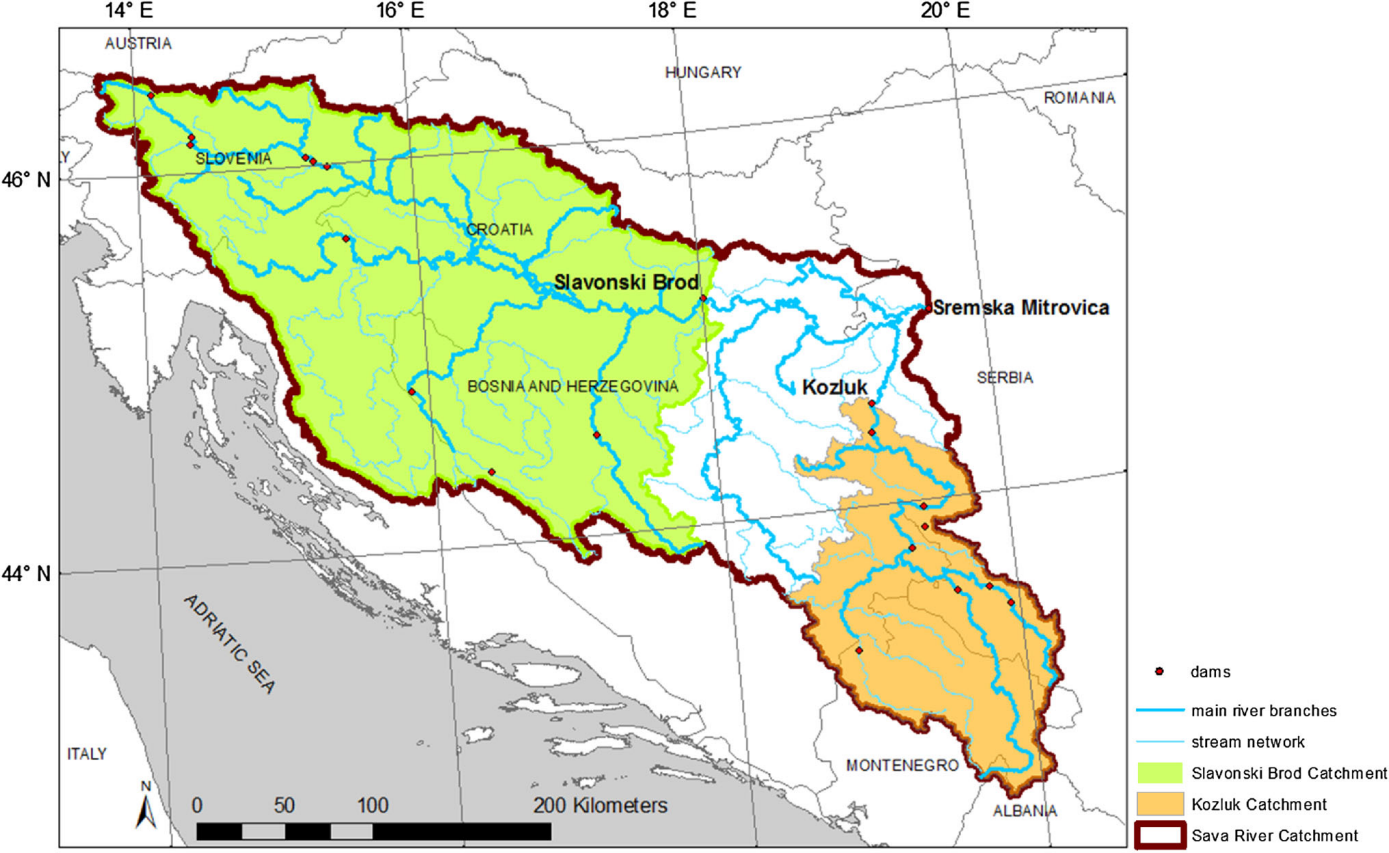
The Sava River Catchment (as defined by the Sremska Mitrovica discharge station) and two investigated subcatchments (Slavonski Brod and Kozluk) within it

In this study, we investigate the hydroclimatic change manifestations and their possible drivers in the major transboundary Sava River Catchment (SRC; Fig. 1), draining into the Danube River in South-Eastern Europe. The SRC represents a world region with relatively limited open accessibility to environmental data, spanning across countries that have undergone recent political and social instability, which also influences the data accessibility situation. For this region, we here use a wide range of sources to compile times series of hydroclimatic data, as well as data on land-use and water-use developments in the catchment over a large part of the twentieth century.

A main aim of this data compilation is to investigate the past-to-present hydroclimatic changes observed in the SRC. A second study aim is to relate the observed water changes in the landscape to various possible change drivers, including atmospheric climate change and historic land/water-use developments in the region; with regard to the latter, watercourses in the SRC have in particular been subject to major regulation for hydropower production and flood protection since the 1950s, indicating the SRC as a possibly useful candidate site for detecting water change effects of these types of developments, in addition to such effects driven by climate change. A third aim is finally to assess the generality of water change results in the SRC, compared to corresponding results in another, climatically different part of the world with similar land/water-use developments.

## Materials and methods

The SRC (Fig. 1) was delineated based on a Digital Elevation Model (DEM) with resolution of 3 arc-second, obtained from the United States Geological Survey (USGS Hydrosheds, US Department of Interior; US Geological Survey 2006) and river network information from the Catchment Characterization and Modelling (CCM) River and Catchment Database (European Commission, Institute for Environment and Ecology 2008). The discharge measurement station that defines the SRC is located at Sremska Mitrovica, located just upstream of Belgrade, with a catchment area of 92 158 km^2^; the Sremska Mitrovica station integrates close to the total SRC discharge into the Danube River, with the total SRC area being 100 095 km^2^ at its outlet into the Danube River.

The SRC extends across six different countries in South-Eastern Europe: Slovenia, Croatia, Bosnia and Herzegovina, Serbia, Monte Negro, and Albania (Fig. 1; the figure shows the SRC part defined by the discharge measurement station Sremska Mitrovica). The Sava River is 990 km long, with its headwaters in Slovenia and its outlet into the Danube River in Belgrade, Serbia. Sava River is the largest tributary by discharge and the SRC is the second largest catchment by area of the Danube River (International Commission for the Protection of the Danube River (ICPDR) 2005).

The SRC population is about 8 176 000 people, representing 46 % of the total population in the main SRC countries (excluding the small SRC parts in Albania and Monte Negro; International Sava River Commission 2008). The SRC elevation ranges from sea level up to 2646 meters. Three different climate types prevail in different parts of the catchment: alpine climate, moderate continental climate, and moderate continental (mid-European) climate.

Temperature and precipitation data for the SRC were downloaded from the Climatic Research Unit Database CGIAR-CSI CRU TS 2.1 (Mitchell and Jones 2005), as time series of monthly *T* and *P* for the period 1901–2002, at 0.5° resolution of the land surface. Based on these datasets, average annual *P* and *T* were calculated for all data grid cells in the SRC. Over the twentieth century part with complete availability of hydroclimatic data, and on average over the whole SRC, the long-term mean annual temperature (*T*) and precipitation (*P*) are 9 °C and 1108 mm year^−1^, respectively (Climatic Research Unit (CRU) 2006; Mitchell and Jones 2005). Sources and time periods of available monthly river discharge data for the Sremska Mitrovica station, from which corresponding runoff (R; discharge normalized with associated catchment area) was calculated, are listed in Supplementary Table S1. Over the same twentieth century part as for the *T* and *P* data, the mean annual *R* is 531 mm year^−1^.

In order to investigate land-use changes between time periods with available hydroclimatic data, we used land cover data downloaded from The Oak Ridge National Laboratory Distributed Active Archive Center (ORNL DAAC; Goldewijk 2010). Table S2 (Supplementary Material) summarizes the land cover conditions for the two time periods with required hydroclimatic data availability: 1931–1960 and 1964–1993. Currently, cultivated land covers 23.2 % of the SRC area, pasture 6.7 %, boreal forest 1.5 %, mixed forest 31.7 %, and deciduous forest 36.1 % (Goldewijk 2010). The SRC area covered by water is 0.8 %.

With regard to parallel water-use conditions, the use of water for irrigation in the SRC is less than 0.299 mm year^−1^ (or 0.03 km^3^ year^−1^), or 0.6 % of the total water-use in the catchment (48 mm year^−1^), and only 0.28 % of the total SRC area is systematically irrigated (International Sava River Commission 2008). For investigation of water-use changes for other purposes than irrigation, we used data for the historical development of hydropower (in terms of normal annual production), which Destouni et al. (2013) showed to be a fruitful proxy measure for characterization of water flux changes related to hydropower developments, as well as surface area and volume of man-made reservoirs from different online sources (as listed in Table S3; Table S2 also summarizes average normal annual production for the same time periods as for the land cover data). Regarding this type of water-use, there are currently 22 water reservoirs within the SRC with volumes greater than 5 × 10^6^ m^3^, among which 16 are used for hydropower production; the largest of the latter contains a water volume of 880 × 10^6^ m^3^.

Historically, hydropower production within the SRC started with the first hydropower plant built in 1898. Since then, 19 large dams (Fig. 1) (i.e., dams with embankment height of more than 15 meters and storage volume exceeding 3 × 10^6^ m^3^) (International Commission on Large Dams 1997) have been built and several more are planned for the near future. Currently, there are 23 hydropower plants (listed and described in Table S3; located next to the dams shown in Fig. 1, with some plants sharing the same water reservoirs) with total normal annual production of 8 × 10^6^ MWh, along with many small and micro-plants (with power capacity range 1–30 MW, and below 1 kW, respectively) (Voros et al. 1999). Along with these plants, flood protection is also provided by multipurpose water reservoirs and dams, with regulation of watercourses, increase of channel cross sections and building of bypass channels, dikes, and detention and retention reservoirs (International Commission for the Protection of the Danube River (ICPDR) 2009).

In addition to analyzing the direct *T*, *P*, and *R* data time series (with average values for the two periods 1931–1960 and 1964–1993 summarized in Table S4), in conjunction with available land-use and water-use data (with average values for the same two periods summarized in Table S4), and in order to distinguish possible climate-driven changes of hydroclimatic conditions in the SRC, actual evapotranspiration (AET) was computed according to two different approaches, the results of which were directly compared. This distinction and comparison methodology follows that used in several previous studies of hydroclimatic change (Shibuo et al. 2007; Asokan et al. 2010; Destouni et al. 2010, 2013; Jaramillo et al. 2013; Van der Velde et al. 2013; Asokan and Destouni 2014; Jaramillo and Destouni 2014).

In the first approach, annual AET_wb_ was computed based on the catchment-scale water balance:

1 $$ {\text{AET}}_{\text{wb}} = P - R - \Delta S, $$

where Δ*S* denotes annual change in water storage over the catchment. Based on the findings of consistent long-term behavior of AET_wb_ for different assumptions of Δ*S* magnitude by Destouni et al. (2013) and Jaramillo et al. (2013), we used the simplest assumption of Δ*S* ≈ 0. Average values of AET_wb_ for the two periods 1931–1960 and 1964–1993 are summarized in Table S4.

In the second approach, for direct comparison with AET_wb_ obtained from Eq. (1), we computed two additional, purely climate-related AET measures, AET_Tclim_ and AET_Bclim_, based on Turc (1954) (Eq. (2)) and Budyko (1974) (Eq. (3)), respectively, as

2 $$ {\text{AET}}_{\text{Tclim}} = \frac{P}{{\sqrt {0.9 + \frac{{P^{2} }}{{PET^{2} }}} }} , $$

3 $$ {\text{AET}}_{\text{Bclim}} = P \cdot \left( {1 - e^{{ - \frac{\text{PET}}{P}}} } \right), $$

where PET is potential evapotranspiration, obtained from Langbein (1949) as,

4 $$ {\text{PET}} = 325 + 21 \cdot T + 0.9 \cdot T^{2} , $$

with mean annual temperature *T* given in °C. For clearer time series comparison of AET variation and change, we also scaled the overall levels of AET_Bclim_ and AET_Tclim_ by multiplication with the ratio of the average AET_wb_ over 1931–1993 and the corresponding average value of AET_Bclim_ and AET_Tclim_, respectively. Resulting multiplication factors for the SRC were then 1.26 for AET_Bclim_ and 1.10 for AET_Tclim_. This scaling was done for easier visual comparison of variability and change around mean evapotranspiration because the unscaled AET_Bclim_ and AET_Tclim_ expressions do not necessarily yield accurate such mean levels across all regions and climates (Destouni et al. 2013; Van der Velde et al. 2014). However, it has been shown that use of a single multiplication factor, calibrating the mean AET_Xclim_ level (where the index X may relate to various methods of estimating AET) to fit a relevant catchment-average level of AET_wb_, can accurately capture the AET variability around its overall average level, temporally as well as spatially within a catchment (Jarsjö et al. 2008).

In addition to the changes in mentioned *T*, *P*, *R*, AET_wb_, AET_Tclim_, and AET_Bclim_, we also investigated changes from 1931–1960 to 1964–1993 in relative evapotranspiration AET_wb_/*P* and in temporal variability of R, in terms of the coefficient of variation of daily *R*, CV(*R*). With the paired two-tailed Student t test, we tested the significance of resulting changes consistently at 0.05 significance level for all variables. The null hypothesis tested was that of no change in the long-term (30-year) average value from 1931–1960 (period 1) to 1964–1993 (period 2), using period 1 as the reference period. The null hypothesis was expressed as: *μ*_1_ = *μ*_2_ = *μ*, where *μ*_1_ and *μ*_2_ are the average values of each investigated variable in period 1 and period 2, respectively, and the alternative hypothesis *μ*_1_ ≠ *μ*_2_ expresses that there is significant change from period 1 to period 2. The hypothesized same average value μ for the two periods was estimated from the available data for the reference period 1.

One aim of this study was also to assess the possible generality of SRC results in relation to corresponding results in a climatically different part of the world with similar types of land/water-use changes. To address this aim, we compared key SRC results of AET_wb_/*P* and CV(*R*) changes with corresponding previous results (Destouni et al. 2013) for a set of 9 Swedish catchments, which are all climatically different from the SRC, and instead representative of colder oceanic, humid continental and subarctic climates. Destouni et al. (2013) grouped and analyzed the Swedish basins in three categories according to their major twentieth-century changes in human land- and water-use: basins with major expansion/intensification of non-irrigated agriculture, basins with major hydropower developments and basins with essentially unregulated rivers and little agriculture. For these groups of Swedish basins, Destouni et al. (2013) found simultaneous increase of AET_wb_/*P* and CV(*R*) under agricultural expansion/intensification in the agricultural basins, AET_wb_/*P* increase and CV(*R*) decrease under hydropower expansion in the hydropower basins and no sustained shifts but only fluctuations in these variables in the unregulated basins with little agriculture. Based on these findings, observed changes in characterisation variables AET_wb_/*P* and CV(*R*) were here used for direct cross-regional comparison in order to address the generality of results for hydroclimatic changes and their possible drivers.

## Results

**Fig. 2:**
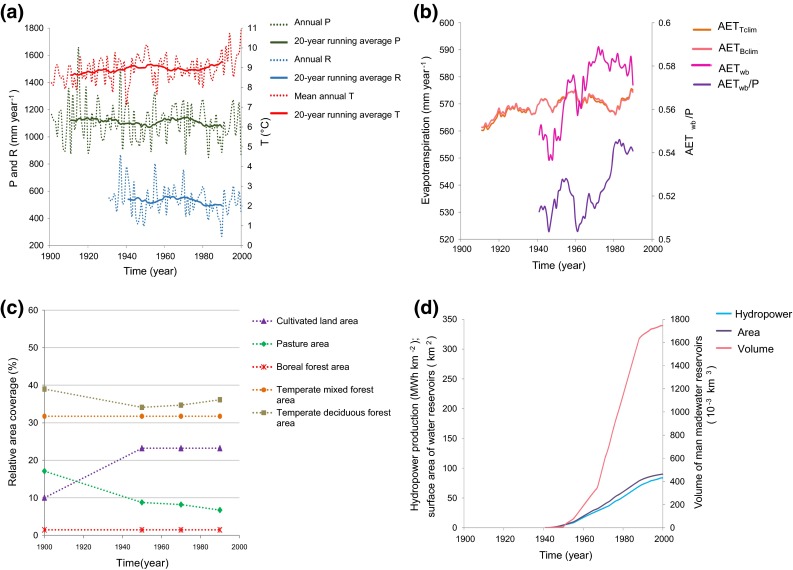
Change and variable co-development within the Sava River Catchment over the twentieth century. **a** Temperature (*T*), precipitation (*P*) and runoff (*R*). **b** Annual average actual evapotranspiration (AET_wb_) and relative actual evapotranspiration (AET_wb_/*P*); results are shown as 20-year moving averages, with AET_wb_ calculated from catchment water balance, and AET_Tclim_ and AET_Bclim_ calculated from Turc and Budyko Eqs. (2) and (3), respectively; the AET_Tclim_ and AET_Bclim_ values shown have been scaled by the ratio of average AET_wb_ in 1931–1993 and corresponding average AET_Tclim_ and AET_Bclim_, respectively. **c** Total area coverage by different land uses. **d** 20-year moving averages of developed annual hydropower production per unit catchment area, water surface area and volume of man-made water reservoirs for the SRC catchment

Figure 2a shows the variability and change in annual and running 20-year annual average of *T*, *P*, and *R* for most of the twentieth century in the SRC. Whereas *T* has increased, *P* and *R* have decreased. With regard to AET_wb_, which for any given *P* conditions also affects *R* (Eq. (1)), Fig. 2b shows the whole AET_wb_ time series in comparison with the purely climate-driven AET_Tclim_ and AET_Bclim_ estimates. A notable increase in AET_wb_ can be seen starting sometime around 1950–1960, with AET_wb_ thereafter remaining at a higher overall level, even though still fluctuating around that level. No corresponding sustained increase can be seen in the purely climate-related AET_Bclim_ and AET_Tclim_ estimates, or in the *T* data (Fig. 2a).

As *P* also shows an increase during the 1950s (Fig. 2a), an important question is if the absolute AET_wb_ shift seen in Fig. 2b is temporally reflected in such a shift also in relative AET_wb_/*P*; the latter would imply an additional change driver beyond the *P* change. Figure 2b shows that a notable shift occurs also in the temporal development of relative evapotranspiration AET_wb_/*P* (purple curve) after 1960.

With regard to possible landscape drivers of this AET_wb_/*P* shift (which is not readily explainable solely by the observed atmospheric climate changes in *T* and/or *P*), Fig. 2c also shows main changes in land-use within the SRC (see also Table S2). These occurred mostly prior to the AET_wb_/*P* shift, with the exception of a 2 % increase in temperate deciduous forest area occurring from year 1950 to 1990. Figure 2d further shows hydropower development in the SRC (normal hydropower production per catchment area, surface area and volume of man-made water reservoirs), with hydropower production capacity shifting from near-zero up to 84 MWh km^−2^ (Tables S2, S3) over the same time that AET_wb_/*P* shifted to its higher level (Fig. 2d).

**Fig. 3:**
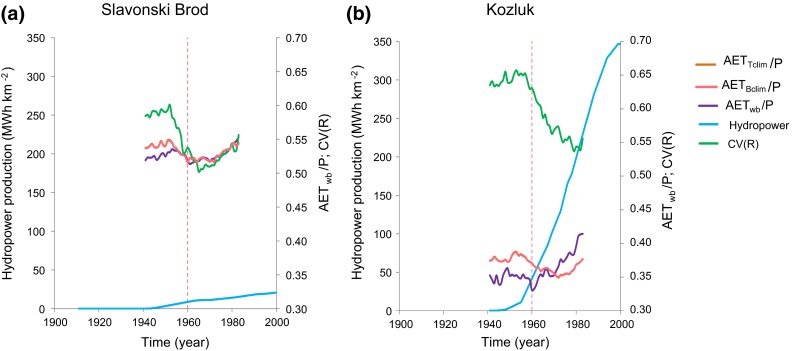
Change and variable co-development over the twentieth century shown as 20-year moving averages for: relative actual evapotranspiration (AET_wb_/*P*); relative evapotranspiration climate estimates of AET_Bclim_/*P* and AET_Tclim_/*P* (where AET_Bclim_ and AET_Tclim_ are calculated from Turc and Budyko Eqs. (2) and (3), respectively; the AET_Bclim_ and AET_Tclim_ values have been scaled by the ratio of average AET_wb_ in 1931–1993 and corresponding average AET_Bclim_ and AET_Tclim_, respectively); developed annual hydropower production per unit catchment area and coefficient of variation of monthly runoff (CV(R)). **a** Slavonski Brod. **b** Kozluk

In order to further investigate the possible co-development of AET_wb_/*P* and hydropower production (as a proxy of also other hydropower-related changes in the SRC, such as those in surface area and volume of man-made water reservoirs; Fig. 2d), we distinguish two subcatchments of the SRC that differ greatly in terms of their hydropower development: Slavonski Brod and Kozluk (Fig. 1). Both Slavonski Brod and Kozluk exhibit similar changes as the whole SRC with regard to increase of *T*, and decrease of *P* and *R* (Fig. S1, Supplementary Material). Similarly to the whole SRC, increase in AET_wb_/*P* is also evident from year 1960 in both catchments (Fig. 3), but with a 2.7 times greater change magnitude in the Kozluk catchment (shift from 0.33 to 0.41) than in the Slavonski Brod catchment (shift from 0.52 to 0.55). Increases are then also visible in the purely climate-related AET_Bclim_/*P* and AET_Tclim_/*P* estimates for the Slavonski Brod catchment (Fig. 3a), but not for the Kozluk catchment where AET_Bclim_/*P* and AET_Tclim_/*P* instead decrease (Fig. 3b); scaling factors for AET_Bclim_ and AET_Tclim_ are 1.35 and 1.18, respectively, for Slavonski Brod, and 0.88 and 0.77, respectively, for Kozluk.

**Fig. 4:**
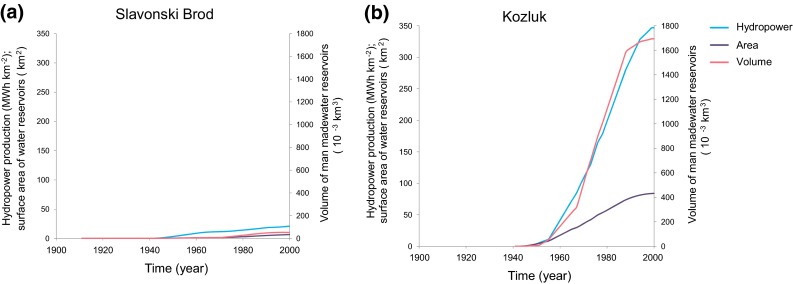
Twenty-year moving averages of developed annual hydropower production per unit catchment area, water surface area and volume of man-made water reservoirs for the SRC catchments. **a** Slavonski Brod. **b** Kozluk

Furthermore, no sustained change in land-use is evident (Fig. S2, Supplementary Material) in any of the two subcatchments. However, the two catchments differ considerably in terms of their developed hydropower production capacity per catchment area (Figs. 3, 4), as well as their surface area and volume of man-made water reservoirs (Fig. 4). Slavonski Brod with catchment area of 54 718 km^2^ has developed normal annual hydropower production of 25 MWh km^−2^, while the corresponding value for Kozluk with a 17 847 km^2^ area is 347 MWh km^−2^ (Tables S2, S3). The Kozluk catchment also exhibits a decrease in its CV(*R*) level from around the year 1960 (Fig. 3b), concurrently with the shift of AET_wb_/*P* to its higher level in this catchment. Around the same time, the Slavonski Brod catchment (Fig. 3a), after a few years of decrease, exhibits instead an increase in CV(*R*).

**Fig. 5:**
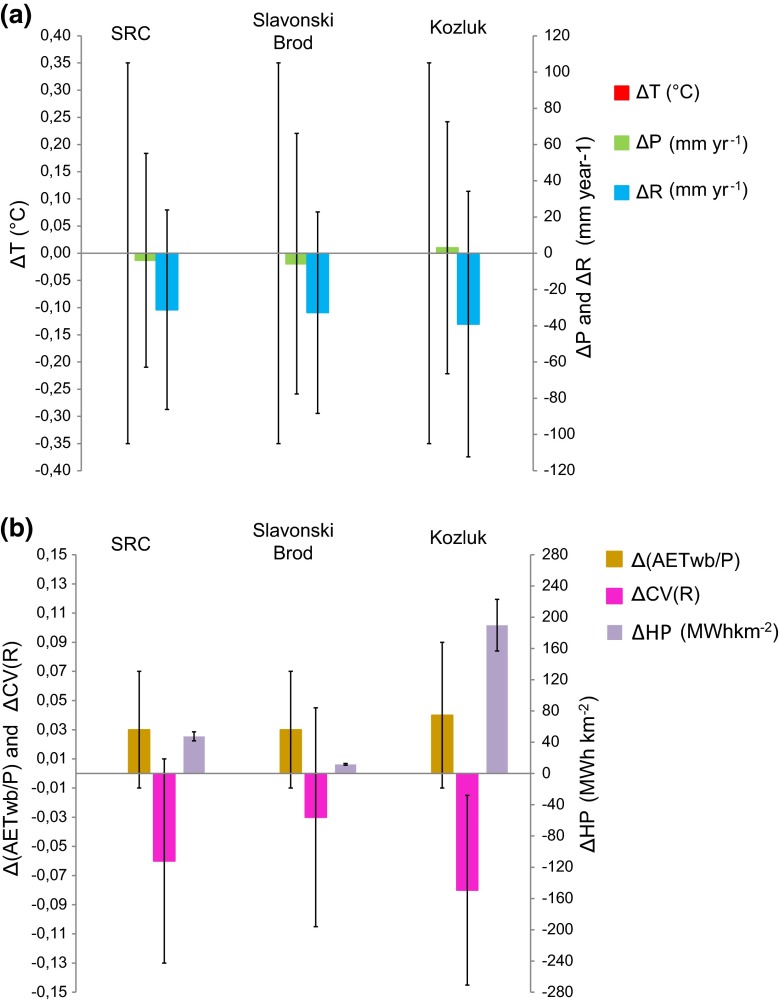
Change (Δ) in hydroclimatic variables and hydropower production development in the SRC and its subcatchments. **a** Temperature (*T*), precipitation (*P*), runoff (*R*). **b** Relative actual evapotranspiration (AETwb/*P*), coefficient of variation of monthly runoff CV(*R*) and developed hydropower production per catchment area (HP). *Error bars* show 95 % confidence intervals for the hydroclimatic and hydropower changes

**Fig. 6:**
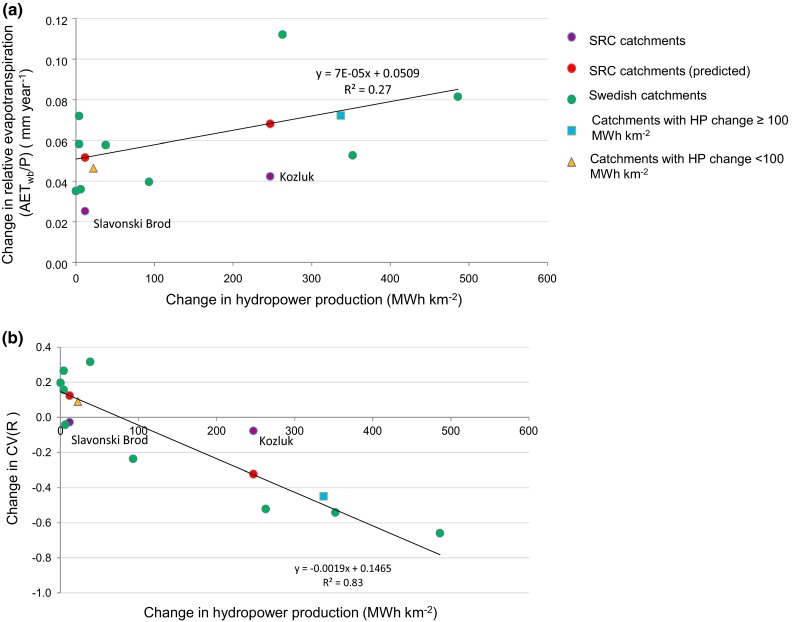
Cross-regional relation between changes. **a** Changes in relative evapotranspiration (AET_wb_/*P*) and hydropower production between periods (1931–1960) and (1971–2000) [(1964–1993) for Slavonski Brod and Kozluk]. **b** Changes in coefficient of variation of runoff CV(R) and hydropower production for the same periods as in **a**. Results are shown for the two different SRC subcatchments (Fig. 1) (*purple symbols*) and compared with previously reported results (Destouni et al. 2013) for different Swedish catchments (*green* *symbols*) and predicted results for the SRC catchments (*red symbols*) calculated on the basis of Swedish catchments results. Regression lines are shown for the Swedish catchments’ results. Illustrated are also values of average AET_wb_/*P* and CV(R) change for the four catchments with hydropower production change of more than 100 MWh km^−2^ (*blue square*) and the seven catchments with less than 100 MWh km^−2^ (*yellow rectangle*)

By the end of the year 1993, 16 times more hydropower production per catchment area (Figs. 4a, b), 13 times more water surface area of man-made reservoirs, and 50 times more water volume were developed in the Kozluk catchment than in the Slavonski Brod catchment. Figure 5a and b summarizes the changes in average T, P, R, AET_wb_/*P*, CV(R), and hydropower production (HP) values from 1931–1960 to 1964–1993, with 95 % confidence interval bars as obtained from the two-tailed paired Student t test for the three catchments. The purely atmospheric climate changes Δ*T* and Δ*P* (Fig. 5a) are not statistically significant for any of the catchments (at 0.05 significance level). However, the decrease in runoff variability ΔCV(*R*) is significant (at 0.05 significance level) only in the Kozluk catchment with the greatest hydropower production per catchment area. In the whole SRC with 4 times lower hydropower production per catchment area (Fig. 5b) and the Slavonski Brod catchment, with 16 times lower hydropower production per catchment area than in the Kozluk catchment, there is no significant change in ΔCV(*R*) (Fig. 5b). For all three catchments, we also performed an ANCOVA test under 0.05 significance level in order to further check the influence of the hydropower production proxy (as an independent variable) on AET_wb_/*P* (as an outcome variable), taking into consideration AET_Bclim_/*P* and AET_Tclim_/*P* (as covariant variables). The test showed highly significant (*P* < 0.001) change in slope of 20-year running average (1940–1983) of AET_wb_/*P* for Kozluk catchment when taking into consideration the influence of hydropower, and no significant change when taking into consideration only climate change. No such significant results were found for Slavonski Brod and the SRC catchments. Figure 6 finally shows a direct comparison between SRC and Swedish (Destouni et al. 2013) catchment results with regard to changes in AET_wb_/*P* (Fig. 6a) and CV(*R*) (Fig. 6b) versus change in developed hydropower production per catchment area. For the Swedish catchments, there is positive correlation for AET_wb_/*P* (*R* ^2^ = 0.27, Fig. 6a) and particularly so for CV(*R*) (*R* ^2^ = 0.83, Fig. 6b). When including the SRC results for the two distinctly different subcatchments Slavonski Brod and Kozluk and removing local noise from the data by considering average results for catchments with hydropower production that is greater (blue square; for 4 such catchments cross-regionally) and smaller (yellow rectangle; for 7 such catchments cross-regionally) than 100 MWh km^−2^, the resulting two average data points fit well to the regression lines for individual catchment data. Figure 6 also shows predicted values for the SRC catchments, calculated on the basis of the Swedish catchment results.

## Discussion

The present results reveal concurrent shifts in relative evapotranspiration AET_wb_/*P* to higher level and runoff variability CV(*R*) to lower level in the SRC hydropower-dominated subcatchment of Kozluk. These AET_wb_/*P* and CV(*R*) shifts are not readily explainable by the observed concurrent climate changes in *T* and *P*, or by any similar changes in the purely *T*- and *P*-dependent estimates of AET_Bclim_/*P* and AET_Tclim_/*P*. At the same time, the Slavonski Brod catchment, with 16 times smaller hydropower production per area than the Kozluk catchment, exhibits no significant increase in CV(*R*), while its increase in AET_wb_/*P* from 1960 is similar to such increases also in AET_Bclim_/*P* and AET_Tclim_/*P* (Fig. 3a).

In other world parts, agricultural expansion, intensification, and irrigation changes have been reported to co-occur with increases in both AET_wb_/*P* and CV(*R*) (Destouni et al. 2013; Jaramillo et al. 2013), but such changes have been small in the SRC. Hydropower production developments have also been reported to co-occur with AET_wb_/*P* increase but then along with a concurrent CV(*R*) decrease (Destouni et al. 2013), such as the changes found here for the Kozluk catchment.

The measure of hydropower production may then be a proxy for various related changes in landscape and atmospheric water. These may, for example, be changes in surrounding soil moisture, groundwater level (decreasing in areas with diverted rivers and increasing close to and downstream of reservoirs) and spatiotemporal variations of temperature (Vercauteren et al. 2013), and associated evapotranspiration (Wildi 2010) induced by the heat storage capacity of created water bodies. Large dams related to hydropower production have indeed also been shown to affect atmospheric water conditions over large distances (up to 100 km) from the actual water reservoirs (Degu et al. 2011) and also snow melt has been shown to be affected by the heat storage capacity of surface water bodies (Vercauteren et al. 2014). All these and other types of water and environmental changes may thus be associated with hydropower-related building of dams, formation of reservoirs, regulation of watercourses, and river/stream/channel diversions and adjustments. Constructed water reservoirs may, further, at least occasionally, also be used for other purposes (industrial, household, flood protection, agricultural), in addition to hydropower production, which can affect regional water conditions and fluxes in various ways.

Different types of changes may thus combine in characteristic ways, quantifiable by the proxy measure of hydropower production, to yield such concurrent increase in AET_wb_/*P* and decrease in CV(*R*) as found here for the most hydropower-affected subcatchment of Kozluk. This hydroclimatic change combination is consistent with similar changes found for comparable hydropower production conditions in Swedish catchments (Destouni et al. 2013) and more recently also in other parts of the world (Jaramillo and Destouni 2014).

## Conclusions

This study has identified two different change signals on the scale of SRC subcatchments. One is related to a dominant hydropower development in the Kozluk subcatchment, which has shifted relative evapotranspiration AET_wb_/*P* to higher level and runoff variability CV(*R*) to lower level. The other signal reflects the essentially unregulated subcatchment of Slavonski Brod, with its increase of AET_wb_/*P* after 1960 being to much greater degree explainable by observed climatic change than in the Kozluk subcatchment, and its CV(*R*) fluctuating around a relatively stable value rather than shifting to some other level. The hydroclimatic change behavior in the whole reflects a combination of these two main subcatchment signals.

Methodologically, this study has shown that it may be possible to find, compile, and synthesize relevant data series for capturing and distinguishing long-term hydroclimatic change even for a complex transboundary catchment like the SRC, in a world region that has recently undergone political and social instability, with generally less accessible environmental data than in Sweden.

Hydroclimatically, the present results have quantitatively framed the recent history and present state of hydroclimate in the SRC, of relevance for water resources in several countries and for a majority of their populations. The present SRC results provide a basis for further assessment and following up of future water resource effects of projected climate and other regional changes. With regard to main drivers of hydroclimatic change, these results are more indicative than conclusive, but their implications are important for understanding water resource development in the region and more generally, and thereby worthy of further investigation and testing across catchments of various scales in different parts of the world.

## Electronic supplementary material

Below is the link to the electronic supplementary material.

Supplementary material 1 (PDF 299 kb)
