# Supplementary material for: Hydroclimatic changes and drivers in the Sava River Catchment and comparison with Swedish catchments
Source: Ambio. 2015 Mar 10;44(7):624–34. doi: 10.1007/s13280-015-0641-0 (PMC4591234; doi:10.1007/s13280-015-0641-0)
Supplement: Supplementary file 1 — Supplementary material 1 (PDF 299 kb) [file 13280_2015_641_MOESM1_ESM.pdf]

Electronic Supplementary Material

This supplementary material has not been peer-reviewed.

Title: **Hydroclimatic changes and drivers in the Sava River Catchment and comparison with Swedish catchments**

Authors: Lea Levi, Fernando Jaramillo, Roko Andričević and Georgia Destouni

Contents

Table S1 Sources and times of daily and monthly discharge data for the studied (sub)catchments according to their outlet station.

Table S2 Annual average area coverage of different land uses, normal annual hydropower production per area (based on information outlined in Table S3), water surface area and volume of man-made water reservoirs (based on information outlined in Table S3), for the Sava River Catchment (for the Sremska Mitrovica station) and two main studied subcatchments (Fig. 1).

Table S3 Information about the 23 hydropower plants in the Sava River Catchment and two main studied subcatchments (Fig. 1). For each of the hydropower plants the following information is given: the catchment to which the plant belongs, the name of the plant, the year that it was built in, the river on which it was built, its normal annual production, surface area and volume of belonging reservoir and a reference number for the source from which the data were obtained, with full references listed below in Electronic Supplementary Material References. The table also summarizes total normal annual hydropower production per catchment area for each catchment in year 2000.

Table S4 Long-term average annual values of the hydroclimatic variables temperature (T), precipitation (P),runoff (R), actual evapotranspiration (AET_{wb}), relative evapotranspiration AET_{wb}/P and coefficient of variation of runoff CV(R) for the Sava River Catchment and two main studied subcatchments (Fig. 1) for two main study time periods.

Figure S1 Change and variable co-development within a) Slavonski Brod catchment and b) Kozluk catchment, over the 20th century. Shown are: Temperature (T), precipitation (P), runoff (R), annual average actual evapotranspiration (AET_{wb}).

Figure S2 Total area coverage by different land uses over the 20th century in the SRC subcatchments. a) Slavonski Brod. b) Kozluk

Electronic Supplementary Material References Numbered as in Table S3

Table S1

Sources and times of daily and monthly discharge data for the studied (sub)catchments according to their outlet station.

Runoff station	Time period	Source
Slavonski Brod	1931-1993	Državni hidrometeorloški zavod Hrvatske (DHMZ), SBrod-Qdn-text document, 2011.
Kozluk	1931-1993	Institute for the development of water resources "Jaroslav Černi", Kozluk-excel document, 2011.
Sremska Mitrovica	1931-2000	Institute for the development of water resources "Jaroslav Černi", SremskaMitrovica-excel document, 2011.

Table S2 Annual average area coverage of different land uses, normal annual hydropower production per area (based on information outlined in Table S3), water surface area and volume of man-made water reservoirs (based on information outlined in Table S3), for the Sava River Catchment (for the Sremska Mitrovica station) and two main studied subcatchments (Fig. 1).

Catchment	Sava River Catchment		Slavonski Brod		Kozluk	
Time period	1931-1960	1964-1993	1931-1960	1964-1993	1931-1960	1964-1993
Cultivated land	23.2	23.0	16.0	16.0	16.0	16.0
Pasture	6.7	7.5	5.9	5.4	5.9	5.4
Boreal forest	1.5	1.5	2.5	2.5	2.5	2.5
Temperate mixed forest	31.7	31.7	23.6	23.6	23.6	23.6
Temperate decidius forest	36.1	35.4	50.8	51.3	50.8	51.3
Hydropower production (MWh km^{-2})	3	50	2	14	3	190
Hydropower production (MWh km^{-2}) in year 2000	84		25		347	
Water surface area of man-made water reservoirs (km^2)	3	58	0.4	3	3	55
Water surface area of man-made water reservoirs (km^2) in year 2000	91		9		84	
Volume of man-made water reservoirs (10^{-3} km^3)	6	1056	2	26	5	1031
Volume of man-made water reservoirs (10^{-3} km^3) in year 2000	1745		55		1693	

Table S3 Information about the 23 hydropower plants in the Sava River Catchment and two main studied subcatchments (Fig. 1). For each of the hydropower plants the following information is given: the catchment to which the plant belongs, the name of the plant, the year that it was built in, the river on which it was built, its normal annual production, surface area and volume of belonging reservoir and a reference number for the source from which the data were obtained, with full references listed below in Electronic Supplementary Material References. The table also summarizes total normal annual hydropower production per catchment area for each catchment in year 2000.

Catchment	Power plant	Built year	River	Annual production MWh $year^{-1}$	Surface area of a belonging reservoir (km^2)	Volume of a belonging reservoir (10^{-3} km^3)	Reference number
Slavonski Brod	Blanca	2009	Sava	144 000	1.38	1.3	1
	Bostanj	2006	Sava	115 000	1.18	1.17	2
	Mavčiče	1986	Sava	62 000	1.00	1.68	3
	Medvode	1953	Sava	72 000	0.72	1.12	4
	Moste	1952	Sava	64 000	0.6	3.07	5
	Vrhovo	1993	Sava	116 000	1.43	1.16	6
	Bočac	1981	Vrbas	307 500	2.33	42.9	7
	Jajce 1	1957	Pliva	232 000	1.1*	4.2*	12
	Jajce 2	1954	Vrbas	175 000	0.12	1.8	12
	Ozalj	1952	Kupa	23 130	0.06	0.55	13
	Una Kostela	1954	Una	50 500	0.14	■	7
Total Slavonski Brod per catchment area	25 MWh km^{-2}						
Kozluk	Bajina Basta	1966	Drina	1 500 000	12.4	218	7
	Bajina Basta reverz	1984	Tara	16 00 000	15	170	7
	Bistrica	1959	Lim	370 000	0.55	4.1	7
	Kokin Brod	1962	Lim	60 000	7.25	209	7
	Mesići	1950	Prača	16 000	■	0.05	8
	Piva/Mratinja	1978	Piva	800 000	12.5	790	7
	Potpeć	1967	Lim	300 000	7	19.8	9
	Tisca	1990	Tisca	10 000	■	■	7
	Uvac	1979	Lim	72 000	6.1	160	10
	Višegrad	1989	Drina	1 370 000	10.07	101	7
	Vrelo Peručac	1927	Vrelo	350	■	■	7
	Zvornik	1955	Drina	500 000	13	21.3	11
Total Kozluk per catchment area	347 MWh km^{-2}						

Sava River Catchment area outside Slavonski Brod and Kozluk catchment	-	-	-	-	-	-
Total Sava River Catchment per catchment area	84 MWh km^{-2}					

*Natural lake

■ Unavailable data

Table S4 Long-term average annual values of the hydroclimatic variables temperature (T), precipitation (P),runoff (R), actual evapotranspiration (AET_{wb}), relative evapotranspiration AET_{wb} and coefficient of variation of runoff CV(R) for the Sava River Catchment and two main studied subcatchments (Fig. 1) for two main study time periods.

Catchment		Sava River Catchment	Slavonski Brod	Kozluk
Area (km^2)		92158	54718	17847
T (°C)	1901-1930	8.7	8.7	8.0
	1931-1960	9.0	9.0	8.3
	1964-1993	9.0	9.0	8.2
P (mm year^{-1})	1901-1930	1120	1227	994
	1931-1960	1105	1181	1038
	1964-1993	1101	1176	1041
R (mm year^{-1})	1931-1960	546	574	676
	1964-1993	514	541	637
AET_{wb} (mm year^{-1})	1931-1960	560	608	362
	1964-1993	582	635	404
AET_{wb}/P	1931-1960	0.51	0.52	0.35
	1964-1993	0.54	0.54	0.39
CV(R)	1931-1960	0.58	0.57	0.63
	1964-1993	0.52	0.54	0.56

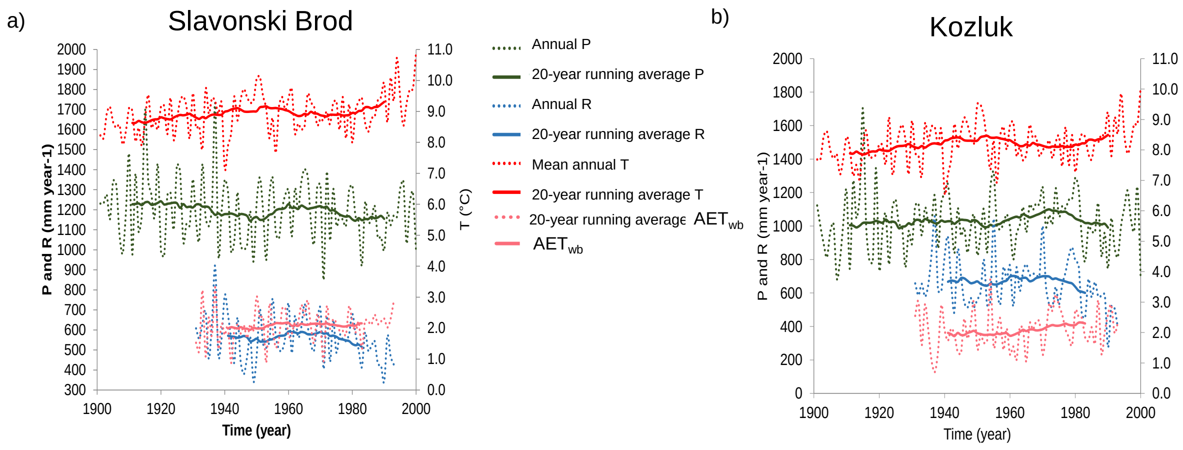

Figure S1 Change and variable co-development within a) Slavonski Brod catchment and b) Kozluk catchment, over the 20th century. Shown are: Temperature (T), precipitation (P), runoff (R), annual average actual evapotranspiration (AET_{wb}).

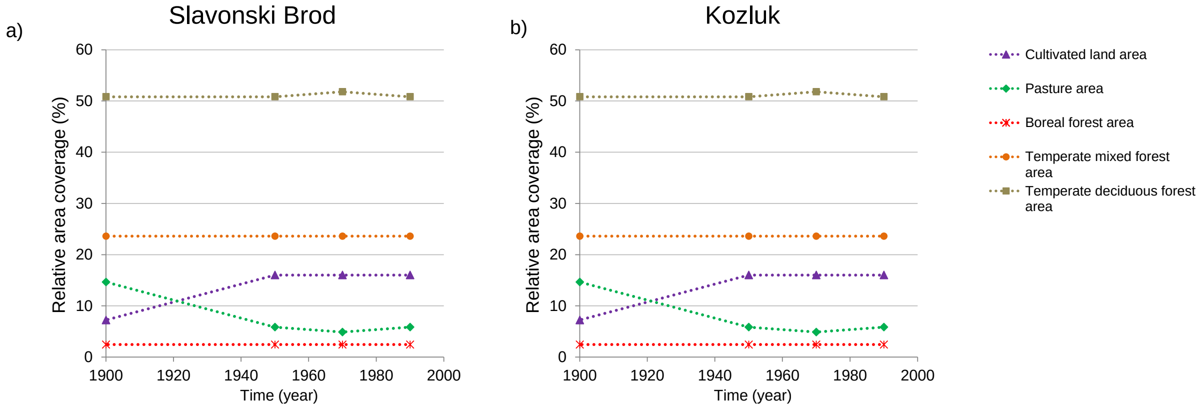

Figure S2 Total area coverage by different land uses over the 20th century in the SRC subcatchments. a) Slavonski Brod. b) Kozluk.

Electronic Supplementary Material References

Numbered as in Table S3

1 Hidroelektrane na spodnji Savi, Retrieved 4 October, 2012, from Hidroelektrane na spodnji Savi web site: http://www.he-ss.si/he-blanca-tehnicne-specifikacije.html

2 Hidroelektrane na spodnji Savi, Retrieved 4 October, 2012, from Hidroelektrane na spodnji Savi web site: http://www.he-ss.si/he-bostanj-tehnicne-specifikacije.html

3 Savske elektrane, Ljubljana d.o.o., 2011, Retrieved 4 October, 2012, from Savske elektrane web site: http://www.sel.si/?p=8&s=2

4 Savske elektrane, Ljubljana d.o.o., 2011, Retrieved 4 October, 2012, from Savske elektrane web site: http://www.sel.si/?p=8&s=3

5 Savske elektrane, Ljubljana d.o.o., 2011, Retrieved 4 October, 2012, from Savske elektrane web site: http://www.sel.si/?p=8&s=1

6 Savske elektrane, Ljubljana d.o.o., 2011, Retrieved 4 October, 2012, from Savske elektrane web site: http://www.sel.si/?p=8&s=4

7 Vučković et al., Postojeće stanje izgrađenosti na slivu Drine, Vodoprivreda, 36, 39-49, 2004.

8 Elektroprivreda Republike Srpske, Retrieved 4 October, 2012, from Elektroprivreda Republike Srpske web site: http://www.ers.ba/stara/distributivne.htm

9 Elektroprivreda Crne Gore AD Nikšić, 2011, Retrieved 4 October, 2012, from Elektroprivreda Crne Gore AD Nikšić web site: http://www.epcg.co.me/01_04_02_02.html

10 Elektro Bijeljina, Retrieved 4 October, 2012, from Elektro Bijeljina web site: http://www.elektrobijeljina.com/Srpski/HTM/MHE_Tisca.htm

11 Elektroprivreda Srbije, Privredno društvo Drinsko-Limske Hidroelketrane Bajina Bašta, 2012, Retrieved 4 October 2012 from Elektroprivreda Srbije web site: http://www.dlhe.rs/he_vrelo.html

12 Hidroelektrane na Vrbasu a.d. Mrkonjić Grad. 2012. Retrieved 4 October, 2012, from Hidroelektrane na Vrbasu a.d. Mrkonjić Grad web site: http://www.henavrbasu.com/hev/Doc.aspx?cat=137&lang=cir&id=195http://www.henavrbasu.com/hev/Doc.aspx?cat=137&subcat=158&id=222&lang=cir&txt=257; http://www.ers.ba/stara/henavrbasu.htm

13 HEP Proizvodnja d.o.o., Retrieved 4 October, 2012, from HEP Proizvodnja d.o.o. web site: http://www.hep.hr/proizvodnja/osnovni/hidroelektrane/zapad/ozalj.aspx

14 SMART (Strategije za poticanje proizvodnje električne energije u malim hidro postrojenjima u Europi). 2009. Male hidroelektrane u Europi: Priručnik o potrebnim administrativnim postupcima.
